# Identification of ibuprofen targeting CXCR family members to alleviate metabolic disturbance in lipodystrophy based on bioinformatics and *in vivo* experimental verification

**DOI:** 10.3389/fendo.2024.1414908

**Published:** 2024-06-26

**Authors:** Zhiwen Cao, Yuxiao Zhao, Ruixin Liu, Xialin Yan, Jiqiu Wang, Na Chen

**Affiliations:** ^1^ Department of Endocrine and Metabolic Diseases, Shanghai Institute of Endocrine and Metabolic Diseases, Ruijin Hospital, Shanghai Jiao Tong University School of Medicine, Shanghai, China; ^2^ Shanghai National Clinical Research Center for Metabolic Diseases, Key Laboratory for Endocrine and Metabolic Diseases of the National Health Commission of the PR China, Shanghai National Center for Translational Medicine, Shanghai, China; ^3^ Department of Colorectal Anal Surgery, The First Affiliated Hospital of Wenzhou Medical University, Wenzhou, China; ^4^ Department of Endocrinology and Metabolism, The First Affiliated Hospital of Wenzhou Medical University, Wenzhou, China

**Keywords:** lipodystrophy, biomarker, ibuprofen, drug therapy, bioinformatic

## Abstract

**Background:**

Lipodystrophy is a rare disease that is poorly diagnosed due to its low prevalence and frequent phenotypic heterogeneity. The main therapeutic measures for patients with clinical lipodystrophy are aimed at improving general metabolic complications such as diabetes mellitus, insulin resistance, and hypertriglyceridemia. Therefore, there is an urgent need to find new biomarkers to aid in the diagnosis and targeted treatment of patients with congenital generalized lipodystrophy (CGL).

**Methods:**

Dataset GSE159337 was obtained via the Gene Expression Omnibus database. First, differentially expressed genes (DEGs) between CGL and control samples were yielded via differential expression analysis and were analyzed for Gene Ontology and Kyoto Encyclopedia of Genes and Genomes enrichment to explore the functional pathways. Next, protein–protein interaction analysis and the MCC algorithm were implemented to yield candidate genes, which were then subjected to receiver operating characteristic (ROC) analysis to identify biomarkers with an area under the curve value exceeding 0.8. Moreover, random forest (RF), logistic regression, and support vector machine (SVM) analyses were carried out to assess the diagnostic ability of biomarkers for CGL. Finally, the small-molecule drugs targeting biomarkers were predicted, and ibuprofen was further validated in lipodystrophy mice.

**Results:**

A total of 71 DEGs in GSE159337 were sifted out and were involved in immune receptor activity, immune response-regulating signaling pathway, and secretory granule membrane. Moreover, CXCR2, TNFSF10, NLRC4, CCR2, CEACAM3, TLR10, TNFAIP3, and JUN were considered as biomarkers by performing ROC analysis on 10 candidate genes. Meanwhile, RF, logistic regression, and SVM analyses further described that those biomarkers had an excellent diagnosis capability for CGL. Eventually, the drug–gene network included ibuprofen–CXCR1, ibuprofen–CXCR1, cenicriviroc–CCR2, fenofibrate–JUN, and other relationship pairs. Ibuprofen treatment was also validated to downregulate CXCR1 and CXCR2 in peripheral blood mononuclear cells (PBMCs) and improve glucose tolerance, hypertriglyceridemia, hepatic steatosis, and liver inflammation in lipodystrophy mice.

**Conclusion:**

Eight biomarkers, namely, CXCR2, TNFSF10, NLRC4, CCR2, CEACAM3, TLR10, TNFAIP3, and JUN, were identified through bioinformatic analyses, and ibuprofen targeting CXCR1 and CXCR2 in PBMCs was shown to improve metabolic disturbance in lipodystrophy, contributing to studies related to the diagnosis and treatment of lipodystrophy.

## 1 Introduction

Lipodystrophy refers to the perturbations on adipose tissue, attributed to the dysfunction or lack of white adipose tissue (WAT). Metabolic syndrome can be identified in the cases of inherited and acquired lipodystrophy, which is also always accompanied by ectopic lipid deposition in striated muscle, liver, and blood. As a result, other comorbidities concerning disorders of lipid and energy metabolism often occur in patients with lipodystrophy, such as diabetes, dyslipidemia, and non-alcoholic fatty liver disease (NAFLD), which may lead to severely advanced clinical outcomes in these patients (1).

WAT is the primary organ in the adult body that physiologically stores lipids, mainly in the subcutaneous and visceral regions. Surplus energy can be stored as glycogen or triglyceride (TD) and released as fatty acid for consumption. Disorder of either storage or catabolism of lipid in WAT would cause metabolic diseases. For instance, obesity is characterized by excessive deposition of TD in adipose tissue. Lipodystrophy occurs when adipocyte differentiation is impaired or excessive fat deposition triggers oxidative stress, resulting in the inability of adipose tissue to store lipid (2). Despite the opposite features in adipose tissue, similar clinical symptoms of metabolic disturbance, such as insulin resistance, hyperinsulinemia, and fatty liver, can be found in both obesity and lipodystrophy (3). Notably, because of the lack of leptin secreted by WAT, patients with lipodystrophy always spontaneously increase their food intake, and abnormal distribution of lipid in multiple organs would aggravate insulin resistance and the disorder of lipid metabolism in the whole body (4). Lipodystrophy is rare and has complex mechanisms; studying patients with lipodystrophy would assist in the profound understanding of lipid metabolism and other metabolic syndromes. Nowadays, the treatment of lipodystrophy is quite similar to other metabolic syndromes, which mainly focuses on alleviating insulin resistance, reducing blood glucose, and facilitating treatment for other metabolic comorbidities that may occur. These treatment strategies, however, seemed to fail to achieve favorable clinical outcomes due to the lack of specificity (4, 5). During the process of lipodystrophy, the adipose tissue of patients continues to demonstrate features of inflammation histologically, including the infiltration of macrophage and other immune cells (6, 7).

Furthermore, the extent of inflammation is reported to be highly related to the severity of insulin resistance in metabolic syndromes (8). Pieces of evidence of systemic inflammation, determined by higher serum levels of interleukins and C-reactive protein, have also been confirmed in patients with insulin resistance. Other researchers also regard inflammation as the “second hit” following the primary driver of insulin resistance in adipose disorder (3, 9, 10). Thus, it is suggested that anti-inflammatory strategies can be the treatment for lipodystrophy. However, as far as we know, there is no proven drug for lipodystrophy to date.

In this study, based on the Gene Expression Omnibus (GEO) database, we first identified differentially expressed genes (DEGs) of congenital generalized lipodystrophy (CGL) in peripheral blood mononuclear cell (PBMC) samples. Subsequent analyses, including random forest (RF), MCC algorithm, and support vector machine (SVM) analysis, were performed to assess the diagnostic capacity of hub genes. Subsequently, we screened the key drug ibuprofen, which may act on two of the hub genes, CXCR1 and CXCR2. We then confirmed the effects of the drug on alleviating metabolic dysfunction in lipodystrophy based on the model of lipodystrophy *in vivo*, thus determining its feasibility as a pharmacological treatment for lipodystrophy.

## 2 Materials and methods

### 2.1 Sources of data

The GSE159337 dataset containing PBMCs for seven CGL samples and PBMCs for seven control samples was obtained via the GEO database (https://www.ncbi.nlm.nih.gov/gds).

### 2.2 Differential expression analysis and enrichment analysis

DEGs between CGL and control samples from the GSE159337 were screened out by R package DESeq2 (version 1.34.0) (11) by setting |log2FC| > 1 and adj. *p* < 0.05, and their expression patterns and chromosomal locations were visualized utilizing the R package OmicCircos (version 1.32.0) (12). After that, Gene Set Enrichment Analysis (GSEA) was implemented based on the gene set “c2.cp.kegg.v7.1.symbols.gmt”, which was downloaded via the Molecular Signatures Database (MSigDB) (https://www.gsea-msigdb.org/gsea/msigdb/index.jsp) with all sorted genes according to log2FC. The top three pathways with positive and negative correlations according to NES sorting in the pathways of *p* < 0.05 were selected for display. In order to explore the functional pathways of DEGs, the Gene Ontology (GO) and Kyoto Encyclopedia of Genes and Genomes (KEGG) enrichment analyses were applied using the R package clusterProfiler (version 4.2.2) (13) by setting *p* < 0.05.

### 2.3 Screening for candidate genes

To explore whether there were interaction relationships among DEGs, a protein–protein interaction (PPI) network (removing the discrete proteins) was constructed via the STRING database (http://string-db.org). After that, DEGs were scored utilizing the MCC algorithm in the Cytohubba software, and the top 10 were selected as candidate genes. Meanwhile, the expression trends of candidate genes between CGL and control samples were analyzed in GSE159337, and the correlation analysis among them was carried out. The functional similarity analysis of the candidate genes was also applied utilizing the R package GOSemSim (14).

### 2.4 Acquisition and evaluation of biomarkers and small-molecule drugs

The candidate genes were subjected to receiver operating characteristic (ROC) analysis, and the genes with area under the curve (AUC) value exceeding 0.8 were screened out as biomarkers. To assess the diagnostic ability of biomarkers for CGL, RF, logistic regression, and SVM analyses were carried out. Based on the biomarkers, the diagnostic model was constructed in GSE159337, and the diagnostic ability of the model was analyzed via confusion matrices and ROC curves. Finally, the small-molecule drugs targeting candidate genes were predicted via the Drug–Gene Interaction Database (DGIdb) (https://www.dgidb.org/), and a drug–gene network was constructed via the Cytoscape software (version 3.8.2).

### 2.5 Construction of lipodystrophy mouse model and ibuprofen treatment

DTRADQ mice were generated using the Cre/LoxP system. In general, R26-(SA-LSL-DTR)flox/flox mice were mated with Adiponectin-cre mice to generate DTRADQ mice, and R26-(SA-LSL-DTR)flox/flox mice were used as control mice. Mice were maintained under a 12-h dark–light cycle with *ad libitum* access to food and water. Body composition was measured using an EchoMRI-100H (EchoMRI). Male mice were used in the experiments. All procedures were approved by the Animal Care Committee of Shanghai Jiao Tong University School of Medicine.

To induce deletion of Cre-positive cells, mice were injected daily with 100 ng/mouse/day of diphtheria toxin (DT) (Biological List Laboratories, Cat#150) for 5 days via the intraperitoneal (i.p.) route. The R26-(SA-LSL-DTR) flox/flox mice received an equal amount of PBS as the control group. Samples were collected at day 7 after the first injection (**Figure 2E**). To determine the effect of ibuprofen on lipodystrophy metabolic dysfunction, 1 mg/mL ibuprofen (Sigma, St. Louis, I1892) was continuously added to the drinking water of both Con + IBU and DTRADQ + IBU groups of mice 7 days prior to intraperitoneal injection until the end of the experiment. After 7 days of IBU pretreatment (D−7 to D0), mice in both the DTRADQ + water and DTRADQ + IBU groups were intraperitoneally injected with DT daily for 5 consecutive days (D0 to D4), and at the same time, mice in the Con + water and Con + IBU groups were injected with an equal volume of PBS. All mice were sacrificed to harvest for tissues on D6 (**Figure 4A**).

### 2.6 Tissue sampling and mouse PBMC isolation

At the end of the experiment, mice were anesthetized with 10% chloral hydrate, and then blood from the orbital plexus was collected in tubes containing EDTA. Blood samples were centrifuged for 10 min at 4,000 rpm, 4°C. The mouse plasma was isolated for subsequent lipid levels. Additionally, epidydimal white adipose tissue (eWAT), inguinal subcutaneous white adipose tissue (iWAT), and the liver of each mouse were precisely dissected, weighed, stored in formaldehyde, snap-frozen in liquid nitrogen, and stored at −80°C for further biochemical testing and analysis.

The Mouse PBMC Isolation Kit (IPHASE, China) was used for analysis according to the instruction. Briefly, fresh anticoagulant whole blood was collected and hemodiluted by adding 1×IPHASE PBMC Washing Buffer at a 1:1 ratio and gently mixed (IPHASE). The diluted blood sample was slowly flattened above the IPHASE Mouse PBMC Isolation Liquid level. The samples were centrifuged at 800 g for 20 min to 30 min at room temperature. The tunica albuginea layer was carefully aspirated into a 15-mL centrifuge tube. Cells were resuspended by adding 10 mL of 1×IPHASE PBMC Washing Buffer Isolation Buffer and centrifuged at 250 g at room temperature for 10 min, and the supernatant was discarded by aspiration and repeated washing to obtain PBMCs. All samples were kept on ice until further RNA extraction.

### 2.7 Lipid measurement

Plasma triglyceride (TG), total cholesterol (TC), low-density lipoprotein cholesterol (LDL-C), and high-density lipoprotein cholesterol (HDL-C) levels were measured with commercial enzymatic reactions kits according to the manufacturer’s guidance (Kehua Bioengineering). Plasma levels of non-esterified fatty acid (NEFA) were determined using a LabAssay™ NEFA kit (FUJIFILM Wako Shibayagi Corporation, LABNEFA-M1) following the instructions.

### 2.8 RNA preparation and real-time qPCR analysis

Total RNA was extracted from PBMCs and liver tissues using the Eastep Super Total RNA Extraction kit (ls1040, Promega Biotech). cDNA was prepared by reverse transcription of 1 μg of total RNA using HiScript III All-in-one RT SuperMix Perfect for qPCR (Vazyme). The ABI system (Life Technologies) was used to perform real-time PCR with ChamQ Universal SYBR qPCR Master Mix (Vazyme), following the manufacturer’s instructions for detection. The housekeeping gene 36B4 was used for analysis using the 2^−ΔΔCT^ method. The primer sequences for the genes are presented in **Supplementary Table S1**.

### 2.9 Morphological analysis

For hematoxylin and eosin (H&E) staining, the adipose and liver tissues were isolated, fixed in 4% paraformaldehyde overnight at room temperature, embedded in paraffin, and sectioned into 5 μm for WAT and liver sections according to standard protocols. IHC was performed with F4/80 (servicebio, GB113373, 1:500) and Ly6G (servicebio, GB11229, 1:500) antibodies in liver tissues. The areas of the positive regions were analyzed with ImageJ. The morphological analysis was conducted using a microscope from Olympus, Japan.

### 2.10 Statistical analysis

Data are reported as mean ± SEM. Statistical analysis was performed using SPSS (IBM SPSS Statistics, Version 22, Armonk, NY). Data that exceeded mean ± twice the standard deviation of its group are regarded as outliers and were omitted. Effects were analyzed using two-way ANOVA, with genotype (Con or DTRADQ) and intervention (ibuprofen or control treatment) as independent variables. When significant effects were present, one-way ANOVA and *post-hoc* Tukey analysis were used to compare groups. Effects were regarded statistically significant when *p* ≤ 0.05.

## 3 Results

### 3.1 Identification of DEGs

**Figure 1:**
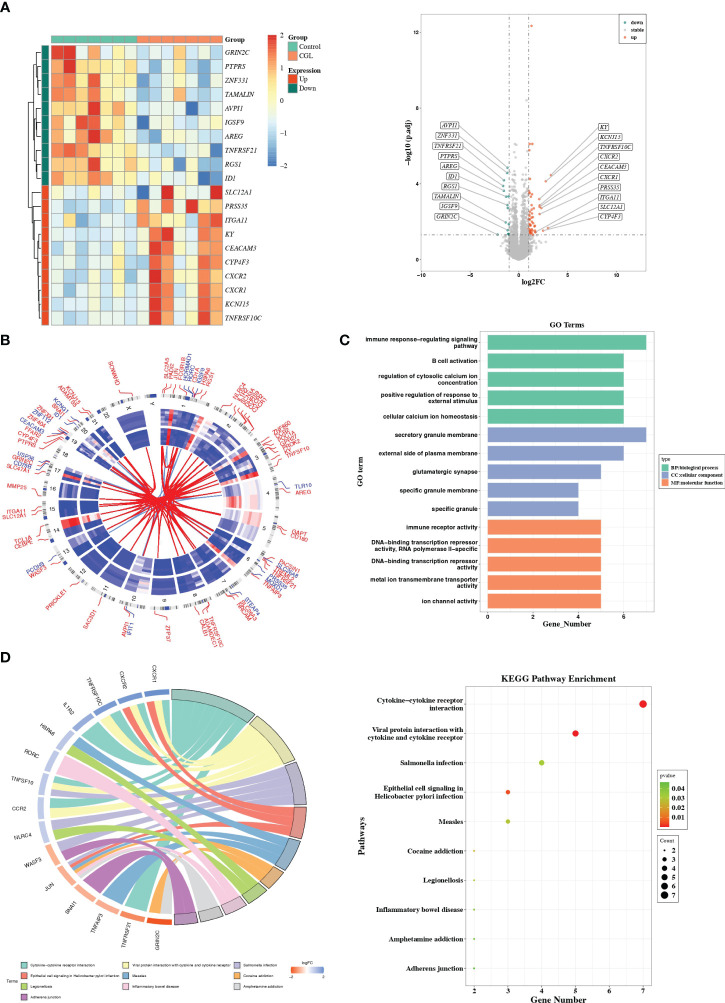
Differential expression analysis and functional studies. **(A)** Heatmap and volcano plot showed the differentially expressed genes in GSE159337. **(B)** Chromosomal mapping. **(C)** The enriched GO terms of DEGs in GSE159337. **(D)** KEGG pathway enrichment results in GSE159337.

There were 71 DEGs between CGL and control samples, and the volcano map and heatmap illustrated the top 10 up- and downregulated genes (**Figure 1A**, **Supplementary Table S2**). The expression patterns and chromosomal locations of DEGs are visualized in **Figure 1B**. The top three pathways with positive NES were systemic lupus erythematosus, galactose metabolism, and tryptophan metabolism, and the top three pathways with negative NES were T-cell receptor signaling pathway, adherens junction, and ribosome (**Supplementary Figures S1A–F**). Moreover, DEGs were involved in GO entries such as immune response-regulating signaling pathways, immune receptor activity, and secretory granule membrane (**Figure 1C**, **Supplementary Figures S1G–I**). Meanwhile, they were involved in KEGG pathways such as cytokine–cytokine receptor interaction, viral protein interaction with cytokine and cytokine receptor, inflammatory bowel disease, adherens junction, and measles (**Figure 1D**).

### 3.2 A total of 10 candidate genes were sifted out

**Figure 2:**
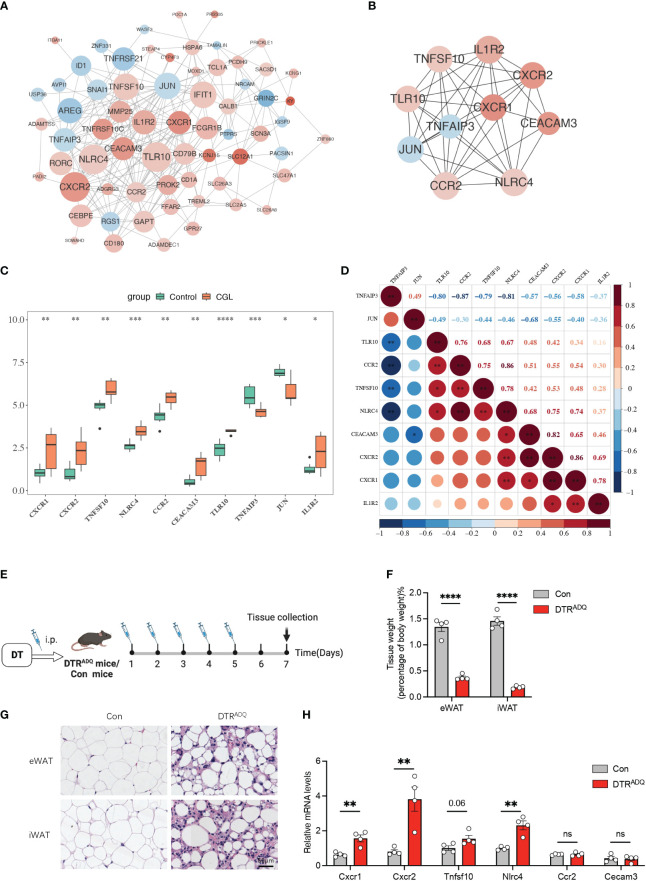
Ten candidate genes were screened by PPI and partially validated in lipodystrophy mouse models. **(A)** The PPI network of the DEGs. **(B)** The PPI network of the 10 hub genes. **(C)** Expression levels of the 10 hub genes between CGL and control samples. **(D)** Correlation analysis of the 10 hub genes. **(E)** Schematic of lipodystrophy mouse experimental design. **(F)** Tissue weights of eWAT and iWAT in con and DTRADQ mice. **(G)** H&E staining of eWAT and iWAT depots from con and DTRADQ mice. Scale bars, 50 mm, *n* = 4 for each group. **(H)** mRNA levels of inflammation-related genes in PBMC from con and DTRADQ mice, *n* = 4 for each group. Data are shown as mean ± SEM. Statistical differences between groups were assessed by unpaired Student’s *t*-test. *p < 0.05; **p < 0.01; ***p < 0.001; ****p < 0.0001. ns, not significant.

The 66 nodes and 247 edges formed a PPI network, including TNFRSF10C, CEACAM3, CXCR1–TLR10, CXCR2–NLRC4, and other reciprocal relationship pairs (**Figure 2A**). Through the MCC algorithm in the Cytohubba software, the 10 candidate genes (CXCR1, CXCR2, TNFSF10, NLRC4, CCR2, CEACAM3, TLR10, TNFAIP3, JUN, and IL1R2) were sifted out (**Figure 2B**), and they were all notably differentially expressed between CGL and control samples (**Figure 2C**). Correlation analysis revealed that CXCR1 was strongly and positively correlated with CXCR2 (Cor = 0.86) (**Figure 2D**). Previous studies have reported CXCR2 and CCR2 as molecular players involved in the development of obesity-induced inflammation and insulin resistance (15). Recently, in the search for new drugs to inhibit pro-inflammatory adipokine secretion in type 2 diabetes patients, emerging evidence has identified that the CXCR1/2 inhibitor ladarixin could enhance insulin resistance in 3T3-L1 adipocytes by reducing inflammation and improving insulin signaling (16). Continuous injection of DT to DTRADQ mice for 5 days can induce adipose loss, which is a commonly used model of lipodystrophy (17). We recorded the first injection as day 1 and assayed mouse WATs on day 7 (**Figure 2E**). Tissue weights were largely decreased in iWAT and eWAT of DTRADQ mice, and H&E staining showed significantly smaller and fewer adipocytes in DTRADQ mice compared to littermates (**Figures 2F, G**). We confirmed the higher expression levels of CXCR1, CXCR2, TNFSF10, and NLRC4 of PBMCs from DTRADQ mice compared to the control mice, while CCR2 and CECAM3 had no significant difference between two groups, which was largely consistent with those of human PBMCs (**Figure 2H**). These data suggested that most of the gene expression changes are the same between human and mouse lipodystrophy, which could help further treatment investigation.

### 3.3 The biomarkers had an excellent diagnosis capability for CGL

**Figure 3:**
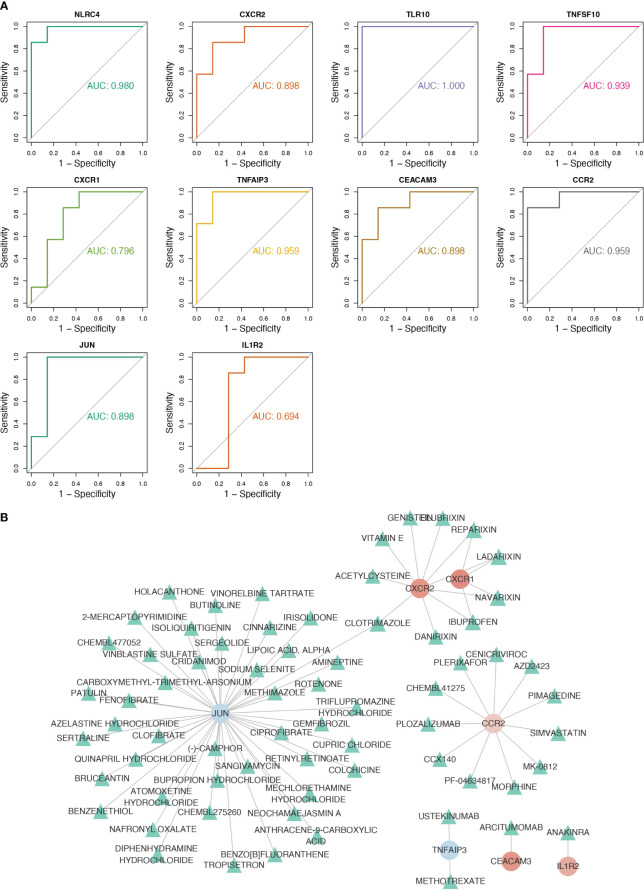
Eight key genes were screened as biomarkers for the diagnosis of CGL, and the drug–gene network was constructed. **(A)** Results of ROC analysis of 10 hub genes. **(B)** The drug–gene network constructed based on 7 out of 10 candidate genes.

NLRC4, CXCR2, TLR10, TNFSF10, TNFAIP3, CEACAM3, CCR2, and JUN were considered as biomarkers because their AUC values all exceeded 0.8 (**Figure 3A**). In RF, logistic regression, and SVM analyses, the confusion matrices showed that the diagnostic model could accurately predict samples from the GSE159337 dataset, and the AUC values of the diagnostic model in these three methods were all greater than 0.7, suggesting that the diagnostic model had an excellent diagnosis capability for CGL (**Supplementary Figures S2A–C**). Eventually, 7 out of 10 candidate genes predicted the drugs and the drug–gene network constructed on the basis of them including ibuprofen–CXCR1, ibuprofen–CXCR2, cenicriviroc–CCR2, fenofibrate–JUN, and other relationship pairs (**Figure 3B**). After drug screening, four drugs—ibuprofen, ladarixin, navarisin, and repamrixin—were found to act on both target genes—CXCR1 and CXCR2—in PBMCs, which prompted us to prioritize the effects of these drugs.

### 3.4 Ibuprofen downregulates CXCR1 and CXCR2 in lipodystrophy PBMCs

**Figure 4:**
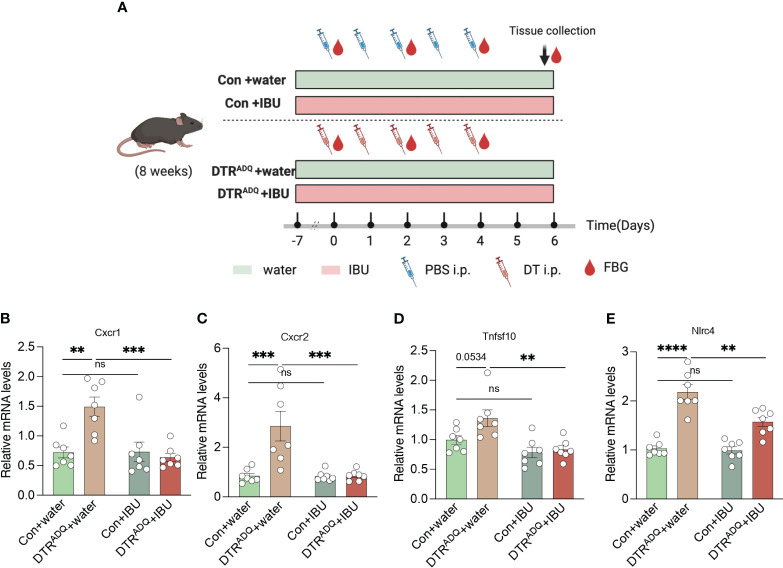
Ibuprofen downregulates the expression of CXCR1 and CXCR2 in PBMC of lipodystrophy mice. **(A)** Schematic of mouse experimental design. **(B–E)** The expression of CXCR1, CXCR2, TNFSF10, and NLRC4 of PBMC in four groups. *n* = 7 for each group. Data are shown as mean ± SEM. Data were analyzed using two-way ANOVA followed by the Tukey *post-hoc* test **(B-E)**. **p < 0.01; ***p < 0.001; ****p < 0.0001. ns, not significant.

To date, there is no cure for lipodystrophy syndrome. Therefore, the current treatment focuses on appetite control and improvement of disorders of glucose-lipid metabolism or other chronic complications. The above findings suggested that four drugs may act on CXCR1 and CXCR2 in PBMCs, in which ibuprofen was reported to improve glucose tolerance in the high-fat diet (18). Therefore, we hypothesized that treatment with ibuprofen would target CXCR1 and CXCR2 in PBMCs and further improve metabolic dysfunction in our mouse model. Ibuprofen treatment began 1 week before DT intraperitoneal injection and continued until the end of the experiment (19, 20). Two groups of mice were divided into four subgroups according to whether the drinking water was supplemented with ibuprofen (IBU) (Con + water, Con + IBU, DTRADQ + water, and DTRADQ + IBU) (**Figure 4A**). As previously found, the expression of CXCR1 and CXCR2 was significantly higher in PBMCs of DTRADQ mice after DT injection than in the control group, and IBU treatment significantly eliminated this elevating effect (**Figures 4B, C**). Meanwhile, ibuprofen did not affect the expression of CXCR1 and CXCR2 in PBMCs of control mice (**Figures 4B, C**) and could not downregulate TNFSF10 and NLRC4 in PBMCs of DTRADQ mice (**Figures 4D, E**), suggesting the specific targeting role of ibuprofen in CXCR1 and CXCR2.

### 3.5 Ibuprofen improves glucose and lipid metabolism in lipodystrophy

**Figure 5:**
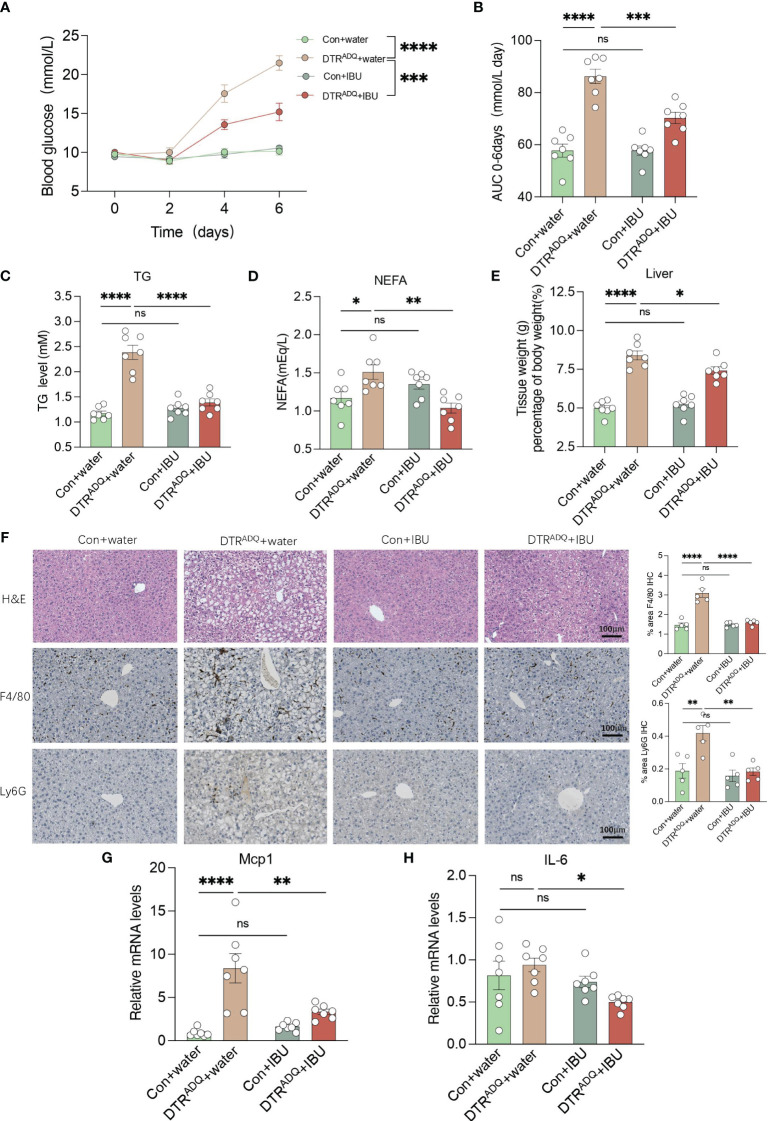
Ibuprofen improves glucose and lipid metabolism, hepatic steatosis, and liver inflammation in lipodystrophy mice. **(A, B)** Fasting blood glucose (FBG) and area under the curve (AUC) plot. *n* = 7 for each group. **(C, D)** Plasma TG and NEFA in four groups. *n* = 7 for each group. **(E)** Tissue weight of liver in four groups. *n* = 7 for each group. **(F)** Representative H&E staining and immunohistochemical staining of F4/80 and Ly6G for liver of the four groups. Bar graph quantified the area of F4/80- and LY6G-positive cells. Scale bars, 100 mm (*n* = 5 for each group). **(G–H**) mRNA levels of Mcp1 and IL6 in liver in four groups. *n* = 7 for each group. Data are shown as mean ± SEM. Data were analyzed using two-way ANOVA followed by the Tukey *post-hoc* test **(A–E, G, H)**. *p < 0.05; **p < 0.01; ***p < 0.001; ****p < 0.0001. ns, not significant.

We next examined whole-body glucose-lipid metabolism. The body weight gain and body composition of mice in the four groups were not affected by IBU treatment (**Supplementary Figures S3A, B**). As expected, DTRADQ mice showed significantly decreased adipose mass and adipocyte number compared to control mice, while there is no difference between two groups with IBU treatment (**Supplementary Figures S3C–E**). We also measured fasting 6-h blood glucose every 2 days after DT treatment and found that DTRADQ mice had gradually increasing fasting blood glucose levels up to 21.5 mmol/L at the end of the experiment (**Figure 5A**), which is consistent with the hyperglycemic symptoms of human lipodystrophy. Surprisingly, ibuprofen treatment significantly reduced blood glucose by over 50% in DTRADQ mice, while having no effect on control mice (**Figures 5A, B**).

Adipose tissue can store excess energy in the form of lipids and reuse it when necessary, and these functions are attenuated or completely lost in lipodystrophy syndromes (21). As a result, excess energy that cannot be deposited in atrophic adipose tissue is released into the bloodstream or deposited in ectopic organs (1). Therefore, we examined the plasma lipid levels, including TG and TC, NEFA, LDL-C, and HDL-C. The plasma TG and NEFA of DTRADQ mice were significantly higher than that of control mice and were greatly improved after IBU treatment, while IBU supplementation had no effect on control mice (**Figures 5C, D**). Consistently, LDL-C and TC had similar trends to TG and there was no difference in HDL-C among the four groups (**Supplementary Figures S3F–H**). Taken together, these results suggested that ibuprofen improves glucose and lipid metabolism in lipodystrophy syndrome.

### 3.6 Ibuprofen ameliorates hepatic steatosis and liver inflammation in lipodystrophy

The liver is a dynamic metabolic organ with a vast blood supply, receiving about a quarter of the blood pumped by the heart. It plays an extremely critical role in many physiological processes, including the regulation of systemic glucose homeostasis and lipid metabolism. We hypothesized that the improvement of CXCR1 and CXCR2 in PBMCs by ibuprofen would probably also have a beneficial ameliorative effect on the inflammatory profile and lipid deposition of fatty liver with circulation. We found that DTRADQ + IBU mice gained a remarkable decrease in liver weight, and consistent results were observed in fatty liver morphology (**Figures 5E, F**). By immunohistochemical staining of the liver for F4/80 and Ly6G as markers of macrophages and neutrophils, we found that ibuprofen could significantly reduce the infiltration of inflammatory cells in the liver of lipodystrophy mice (**Figure 5F**). Furthermore, gene expression analysis showed that ibuprofen remarkably decreased the expression of Mcp1 and IL-6 in the lipodystrophy group (**Figures 5G, H**). These data suggested that ibuprofen treatment significantly reduced lipid deposition and inflammation in the liver of lipodystrophy mice.

## 4 Discussion

Lipodystrophy is a combination of complex conditions, mirroring the obesity-related metabolic syndrome. The main characteristics include reduced adipose tissue mass, as well as its capacity to store surplus energy. The key genes that may drive the development of inherited lipodystrophy are yet to be discovered. Adipose transplantation is one of the most effective methods to increase fat mass to treat lipodystrophy but remains challenging in clinical practice just like the other transplantation procedures because of the large shortage of donors and technical difficulties. Intriguingly, because of the similar metabolic sequelae, there are shared treatment strategies for both obesity and lipodystrophy, which focus on enhancing adipocyte function and alleviating lipid overload, to improve the metabolic syndrome of the whole body. Therefore, investigations of etiology and treatment of lipodystrophy may benefit both lipodystrophy and obesity-related metabolic disease. In the current study, based on the GEO database of PBMC RNA-Seq of CGL, a heterogeneous autosomal recessive disorder, we herein reported the eight key hub genes of lipodystrophy, whose diagnostic capacity was also analyzed. Ibuprofen, which was identified to act on at least two hub genes, was screened as the potential pharmacological treatment.

Among the 71 DEGs that were screened between CGL and the control group, we identified genes related to inflammatory regulation, including members of tumor necrosis factor receptor superfamily and IL receptor. This may explain the results of GO and KEGG pathway analysis, which contains the biological process of immune response-regulating signaling pathway and immune receptor activity, among others. These results partially support the perspective that the pathogenesis of adipose disorders is the result of altered infiltration of immune cells as well as the abnormalities of inflammatory cytokines in adipose tissue (22, 23). Further analysis by MCC algorithm and ROC curve identified eight key hub genes (CXCR2, TNFSF10, NLRC4, CCR2, CEACAM3, TLR10, TNFAIP3, and JUN), with an AUC higher than 0.8 for diagnostic purposes for CGL in internal validation. Among the identified hub genes, CXCR1 and CXCR2 encode the proteins of the G-protein-coupled receptor family, which are known as interleukin 8 receptor type 1 and type 2 (IL8RA and IL8RB). It is also reported that cells with ASC (adipose stromal cell) phenotypes, featuring increased CXCR1 and CXCR2 expression, can become mobilized from WAT and infiltrate tumors in obese patients (24). Toll-like receptors (TLRs) share a similar cytoplasmic signaling domain of IL-1, and TLR10 is the only TLR that exerts an anti-inflammatory effect (25). In human adipose tissue, individuals with TLR10 gene polymorphisms were proved to exhibit a reduced infiltration of macrophage in obese individuals and may result in the TLR10-induced inhibition of inflammation during obesity (26). It is demonstrated that the transcription of Tumor Necrosis Factor α-Induced Protein 3 (TNFAIP3) is altered, leading to the promotion of metabolic inflammation through the ablation of GPSM1 in a mouse model of high-fat diet-induced insulin resistance (27). Recent studies reported that TNFAIP3 could block the onset and progression of nonalcoholic steatohepatitis (NASH) by suppressing ASK1 hyperactivation (28) or inhibiting TAK1 activation and was also regarded as the promising therapeutic target of NASH (29). Notably, both NASH and insulin resistance are typical metabolic disorders of lipodystrophy (28, 29), indicating that targeting these key hub genes of lipodystrophy may also help alleviate the metabolic comorbidities of lipodystrophy. Taken together, based on these results, for the first time, we revealed the intimate association between CGL and systematic inflammatory or immune regulations. In addition, these identified key genes could also serve as potential targets for anti-inflammatory therapy in adipose or liver tissues in the management of CGL.

Moreover, through analysis using the DGIDB database, we identified drugs that may target the genes mentioned above. Five drugs were found to act on two target genes, prompting us to initially assess the potential of these five drugs in treating lipodystrophy (**Figure 3B**). Reparixin, a chemokine receptor inhibitor targeting CXCR1 and CXCR2, is recognized for its anti-inflammatory effects. It is predominantly used for treating conditions such as cancer, sepsis, ischemia–reperfusion injury, and hypertension. A multicenter, randomized, double-blind, placebo-controlled trial on newly diagnosed type 1 diabetes revealed that short-term ladarixin treatment did not significantly preserve residual β-cell function (30). Navarixin is currently mainly used in the treatment of advanced solid tumors, and there are no experiments investigating the relationship between navarixin and metabolic diseases (31). Clotrimazole is a broad-spectrum antifungal agent that is mainly used to treat fungal infections such as *Candida albicans*. Clotrimazole stimulated insulin secretion from rat islets incubated with 6 mM glucose in a dose-dependent manner (32).

Ibuprofen, a widely used non-steroidal anti-inflammatory drug (NSAID), is commonly prescribed for fever reduction in both adults and children. Past reports have underscored NSAIDs’ potential effects on metabolic disorders; for example, aspirin has been linked to a decreased risk of diabetes in the elderly and improved glucose metabolism (33). The anti-inflammatory properties of aspirin provide a theoretical basis for exploring ibuprofen’s potential to ameliorate glucose and lipid metabolism in lipodystrophy. A 2008 study indicated that over-the-counter pain relievers, including ibuprofen, improved glucose tolerance in mice on a high-fat diet (34). Our findings indeed demonstrate that ibuprofen can enhance glucose and lipid metabolism, alleviate hepatic steatosis, and reduce liver inflammation in lipodystrophy mice (18). Based on this, we propose that drugs for treating conditions like lipodystrophy may extend beyond ibuprofen, potentially encompassing other NSAIDs. Furthermore, our results suggest that ibuprofen may exert beneficial effects in other diseases such as diabetes and fatty liver.

In the current study, we identified eight biomarkers associated with the diagnosis and treatment of CGL. For the first time, our research revealed that ibuprofen downregulates CXCR1 and CXCR2 in PBMCs of lipodystrophy, leading to improvements in glucose and lipid metabolism associated with lipodystrophy. Additionally, ibuprofen demonstrated positive effects on hepatic fat accumulation and liver inflammation. Our research also suggested the therapeutic potentials of other drugs targeting CXCR family members, such as reparixin, as well as other NSAIDs for lipodystrophy. The limitations of the study include the fact that only mouse models, and not the clinical data of patients with lipodystrophy, were used to validate the therapeutic effects. In addition, we only investigated the metabolic improvement of ibuprofen in male lipodystrophic mice; whether female lipodystrophic mice respond to ibuprofen in the same way remains to be further explored and validated. Moreover, this study did not observe ibuprofen’s ability to induce recovery of WAT *in vivo*. Further studies should focus on the potential synergistic effects of ibuprofen, as well as other NSAIDs or drugs targeting CXCR family members, in combination with current treatments for lipodystrophy (metreleptin, etc.), in order to achieve better therapeutic strategies for lipodystrophy.

## Data availability statement

The datasets analyzed during the current study are available in the GEO database (https://www.ncbi.nlm.nih.gov/geo/). This data can be found here: NCBI, GSE159337, https://www.ncbi.nlm.nih.gov/geo/query/acc.cgi?acc=GSE159337.

## Ethics statement

Ethical approval was not required for the studies on humans in accordance with the local legislation and institutional requirements because only commercially available established cell lines were used. The animal study was approved by the Animal Care Committee of Shanghai Jiao Tong University School of Medicine. The study was conducted in accordance with the local legislation and institutional requirements.

## Author contributions

ZC: Investigation, Methodology, Writing – original draft. YZ: Investigation, Methodology, Writing – original draft. RL: Conceptualization, Project administration, Supervision, Writing – review & editing. XY: Data curation, Methodology, Writing – review & editing. JW: Conceptualization, Project administration, Supervision, Writing – review & editing. NC: Conceptualization, Project administration, Supervision, Writing – original draft.
