# Supplementary material for: Identification of ibuprofen targeting CXCR family members to alleviate metabolic disturbance in lipodystrophy based on bioinformatics and in vivo experimental verification
Source: Front Endocrinol (Lausanne). 2024 Jun 26;15:1414908. doi: 10.3389/fendo.2024.1414908 (PMC11236084; doi:10.3389/fendo.2024.1414908)
Supplement: Supplementary file 1 [file DataSheet_1.docx]

Supplementary Material

**Supplementary Figure 1.** Results of GSEA and GO enrichment analysis.

A-F, Results of GSEA enrichment analysis. G-I, Biological processes, cellular components, and molecular functions of differentially expressed genes.


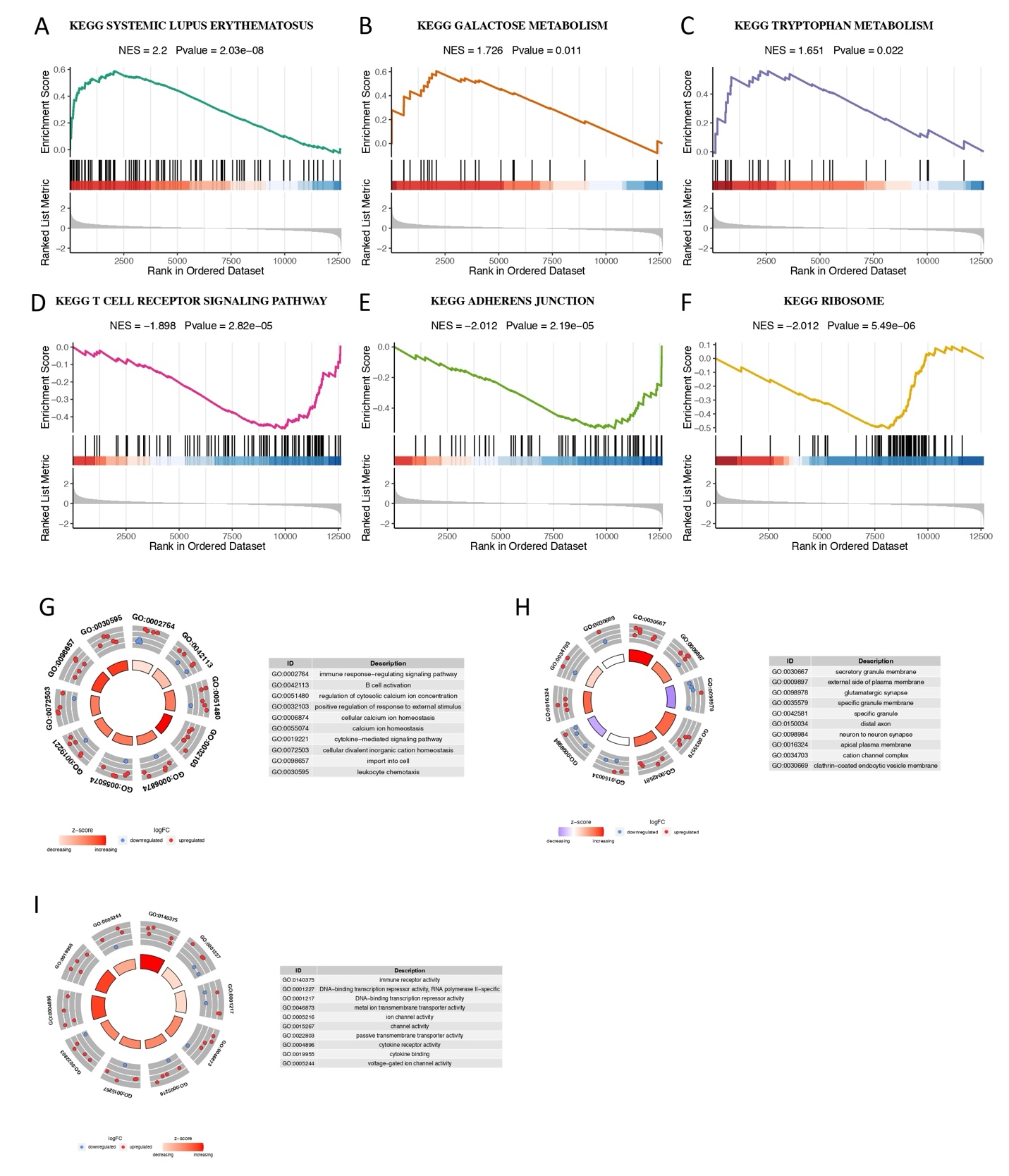


**Supplementary Figure 2.** The diagnostic model in three methods.

A-C, RF, Logistic and SVM Model Confusion Matrix and ROC curve.


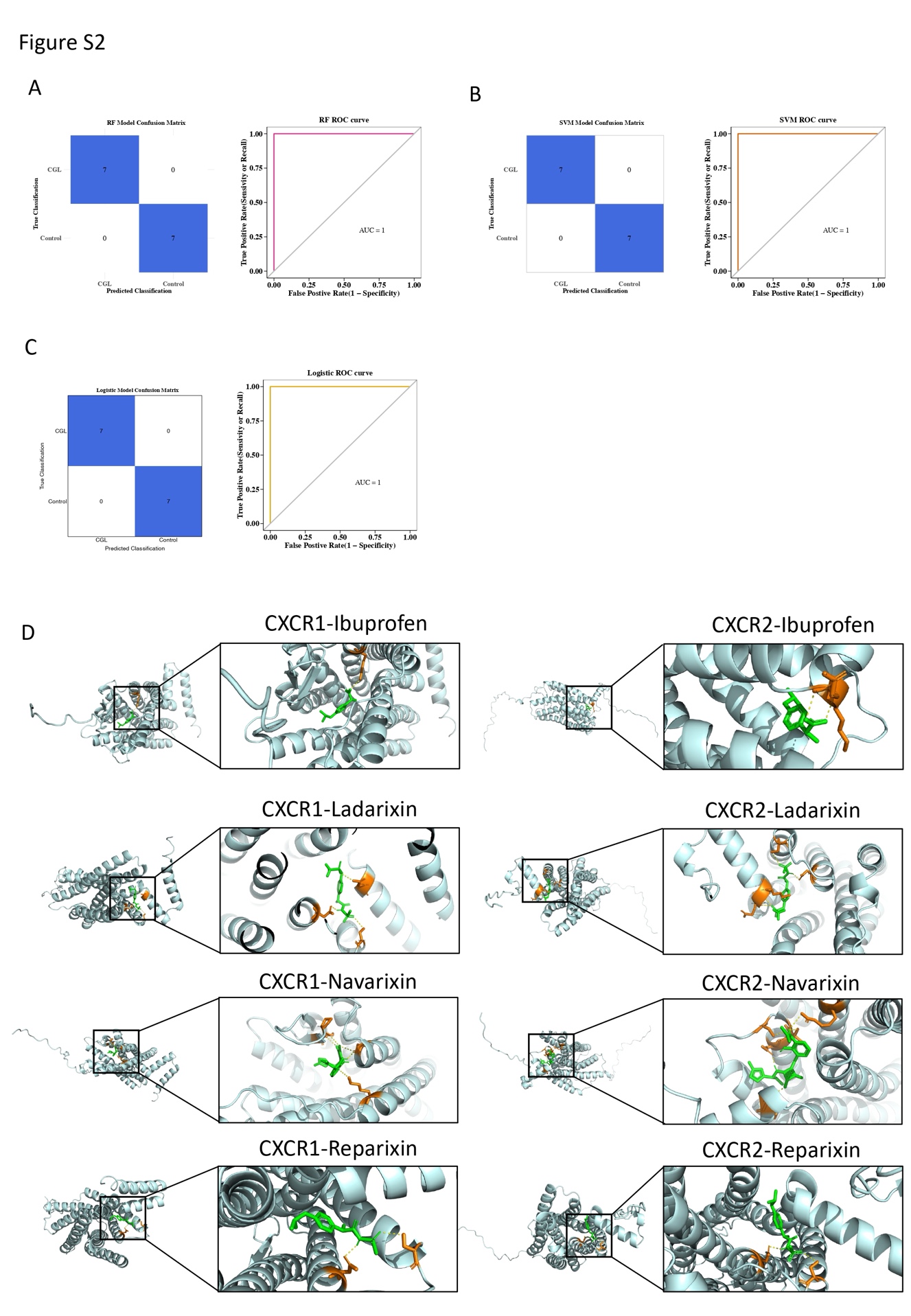


**Supplementary Figure 3.** Ibuprofen had no effect on body weight gain, body composition, fat mass, and HDL-C.

A-E, Body weight (A), body composition (B), tissue weight (C, D), and H&E staining of iWAT and eWAT (E) in four groups of mice, n = 7 for each group. F-H, Plasma TC, LDL and HDL in four groups of mice, n = 7 for each group. Data are shown as mean ± S.E.M.. Data were analyzed using two-way ANOVA followed by the Tukey post hoc test (A to E, G to H). * P < 0.05; ** P < 0.01; *** P < 0.001; **** P < 0.0001.


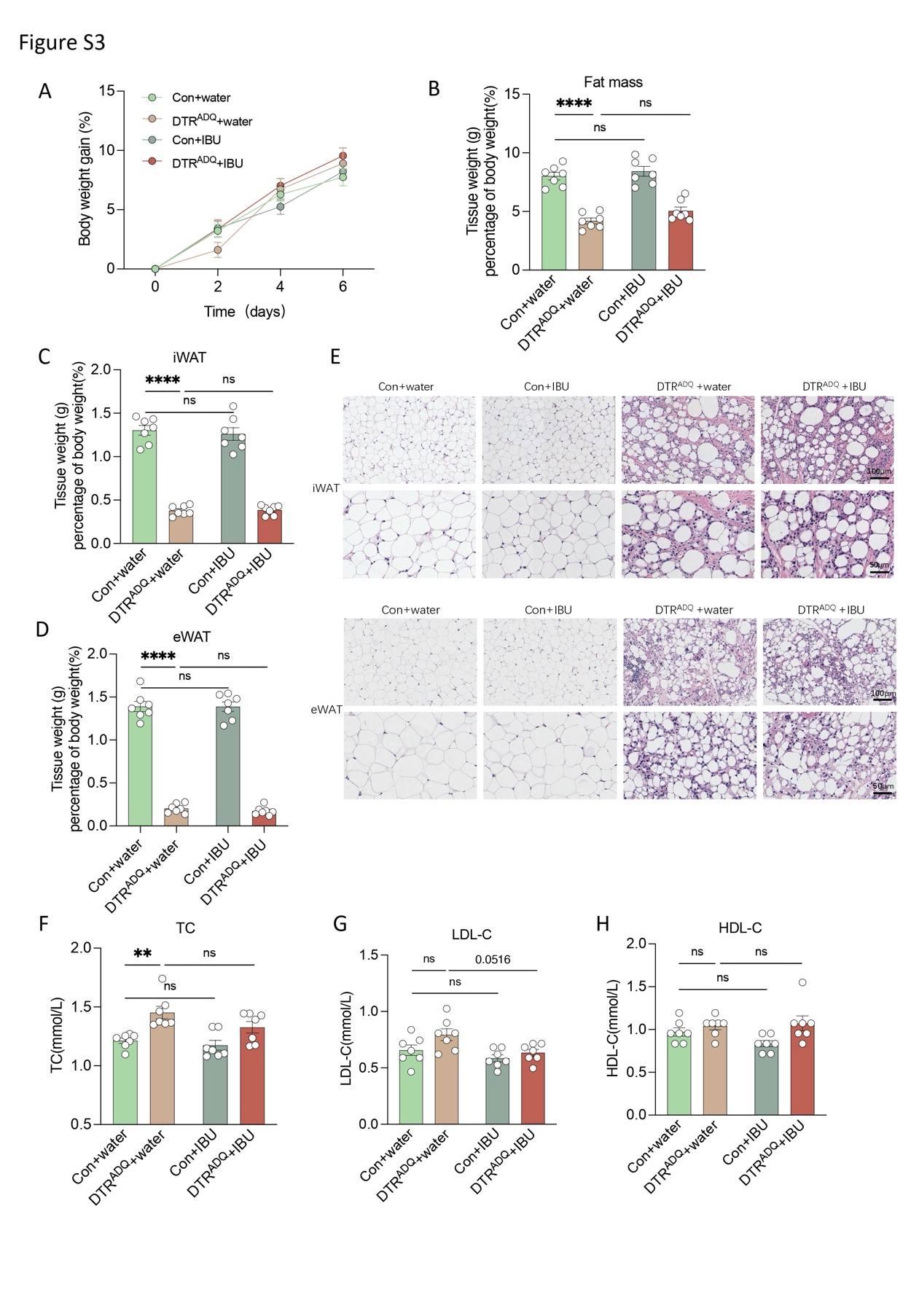


**Supplementary Table 1.** The primers for qPCR.

| **qPCR Primers** | **sequence (5’→3’)** |
| --- | --- |
| 36b4-F | GAAACTGCTGCCTCACATCCG |
| 36b4-R | GCTGGCACAGTGACCTCACACG |
| Cxcr1-F | CCATTCCGTTCTGGTACAGTCTG |
| Cxcr1-R | GTAGCAGACCAGCATAGTGAGC |
| Cxcr2-F | CTCTATTCTGCCAGATGCTGTCC |
| Cxcr2-R | ACAAGGCTCAGCAGAGTCACCA |
| Tnfsf10-F | GGAAGACCTCAGAAAGTGGCAG |
| Tnfsf10-R | TTTCCGAGAGGACTCCCAGGAT |
| Nlrc4-F | CTCACCACGGATGACGAACAGT |
| Nlrc4-R | TGTCATCCAGTATGAGTCTCTCG |
| Ccr2-F | GCTGTGTTTGCCTCTCTACCAG |
| Ccr2-R | CAAGTAGAGGCAGGATCAGGCT |
| Ceacam3-F | TGCCCACCACTACCCAAGT |
| Ceacam3-R | CCCCTTTGTACCAGATAAAGCC |
| Mcp1-F | GGAATTCACCACCATGCAGGTCCCTGTC |
| Mcp1-R | GCGGATCCGAGTCACACTAGTTCACT |
| IL6-F | AGTTGCCTTCTTGGGACTGA |
| IL6-R | TCCACGATTTCCCAGAGAAC |

**Supplementary Table 2.**

| baseMean | log2FoldChange | lfcSE | stat | pvalue | padj |  |
| --- | --- | --- | --- | --- | --- | --- |
| SLC2A5 | 83.04873 | 1.176976 | 0.325644 | 3.614303 | 0.000301 | 0.014473 |
| PADI2 | 86.11282 | 1.403409 | 0.422886 | 3.318649 | 0.000905 | 0.029465 |
| JUN | 3077.537 | -1.02854 | 0.282365 | -3.64261 | 0.00027 | 0.013455 |
| FCGR1B | 28.42222 | 1.434049 | 0.364978 | 3.929142 | 8.52E-05 | 0.005762 |
| HORMAD1 | 26.10247 | 1.104478 | 0.288689 | 3.825843 | 0.00013 | 0.007807 |
| RORC | 25.7075 | 1.098076 | 0.307638 | 3.569375 | 0.000358 | 0.01621 |
| CD1A | 22.21753 | 1.309075 | 0.310245 | 4.219491 | 2.45E-05 | 0.002334 |
| IGSF9 | 19.15622 | -1.16057 | 0.34777 | -3.33719 | 0.000846 | 0.028148 |
| HSPA6 | 81.95959 | 1.129852 | 0.272997 | 4.138703 | 3.49E-05 | 0.002983 |
| RGS1 | 1126.898 | -1.17851 | 0.267457 | -4.40634 | 1.05E-05 | 0.001329 |
| NLRC4 | 264.3083 | 1.029134 | 0.214432 | 4.799352 | 1.59E-06 | 0.000324 |
| IL1R2 | 47.22256 | 1.685321 | 0.520256 | 3.239407 | 0.001198 | 0.036324 |
| SCN3A | 263.9676 | 1.464123 | 0.39795 | 3.67916 | 0.000234 | 0.012121 |
| DHRS9 | 38.00031 | 1.533333 | 0.358559 | 4.276372 | 1.90E-05 | 0.001984 |
| CXCR2 | 78.8898 | 2.139134 | 0.487344 | 4.389373 | 1.14E-05 | 0.001368 |
| CXCR1 | 76.69095 | 2.303263 | 0.536904 | 4.2899 | 1.79E-05 | 0.001903 |
| ZNF860 | 95.1639 | 1.06236 | 0.322335 | 3.295828 | 0.000981 | 0.031242 |
| CCR2 | 788.6342 | 1.048965 | 0.259969 | 4.034957 | 5.46E-05 | 0.004158 |
| POC1A | 16.04715 | 1.160377 | 0.346788 | 3.346074 | 0.00082 | 0.027479 |
| GPR27 | 62.01418 | 1.355038 | 0.283878 | 4.773315 | 1.81E-06 | 0.000358 |
| PROK2 | 84.18307 | 1.602259 | 0.453223 | 3.535257 | 0.000407 | 0.017827 |
| KY | 25.05458 | 3.283413 | 0.605292 | 5.424508 | 5.81E-08 | 3.47E-05 |
| TNFSF10 | 850.331 | 1.096059 | 0.285663 | 3.836901 | 0.000125 | 0.007535 |
| TLR10 | 268.1035 | 1.140886 | 0.214188 | 5.326565 | 1.00E-07 | 5.50E-05 |
| AREG | 282.9969 | -1.54666 | 0.315858 | -4.89671 | 9.75E-07 | 0.000234 |
| GAPT | 374.235 | 1.099346 | 0.174703 | 6.292674 | 3.12E-10 | 7.89E-07 |
| CD180 | 235.6323 | 1.293666 | 0.153545 | 8.425292 | 3.60E-17 | 4.55E-13 |
| PACSIN1 | 11.28893 | -1.07512 | 0.340888 | -3.15387 | 0.001611 | 0.044078 |
| SLC26A8 | 9.885074 | 1.29752 | 0.402346 | 3.224886 | 0.00126 | 0.037478 |
| TREML2 | 168.2068 | 1.065597 | 0.174108 | 6.12034 | 9.34E-10 | 1.69E-06 |
| TNFRSF21 | 24.56005 | -1.53627 | 0.29316 | -5.24037 | 1.60E-07 | 6.53E-05 |
| PRSS35 | 8.671667 | 2.132062 | 0.526716 | 4.047843 | 5.17E-05 | 0.00396 |
| MOXD1 | 20.92058 | 1.250714 | 0.392481 | 3.186688 | 0.001439 | 0.040995 |
| TNFAIP3 | 1603.904 | -1.05492 | 0.224531 | -4.69831 | 2.62E-06 | 0.000468 |
| STEAP4 | 207.0734 | 1.154538 | 0.357524 | 3.229259 | 0.001241 | 0.037084 |
| SLC26A3 | 11.28963 | 1.341341 | 0.357974 | 3.747038 | 0.000179 | 0.010051 |
| NRCAM | 112.6342 | -1.13362 | 0.264284 | -4.2894 | 1.79E-05 | 0.001903 |
| TNFRSF10C | 41.49632 | 2.097419 | 0.456074 | 4.598861 | 4.25E-06 | 0.000639 |
| ADAMDEC1 | 23.87003 | 1.077214 | 0.270208 | 3.986606 | 6.70E-05 | 0.004868 |
| CALB1 | 22.05013 | 1.001884 | 0.293178 | 3.417322 | 0.000632 | 0.023627 |
| ZFP37 | 54.6136 | 1.022249 | 0.209127 | 4.888168 | 1.02E-06 | 0.000237 |
| IFIT1 | 242.8341 | 1.137008 | 0.348521 | 3.262379 | 0.001105 | 0.034478 |
| AVPI1 | 64.38597 | -1.1717 | 0.206277 | -5.6802 | 1.35E-08 | 1.42E-05 |
| SAC3D1 | 20.71485 | 1.008769 | 0.262797 | 3.838584 | 0.000124 | 0.007535 |
| PRICKLE1 | 36.79255 | 1.229721 | 0.26314 | 4.67325 | 2.96E-06 | 0.00048 |
| TAMALIN | 256.699 | -1.35707 | 0.365275 | -3.71519 | 0.000203 | 0.010967 |
| WASF3 | 16.54736 | -1.0118 | 0.326524 | -3.0987 | 0.001944 | 0.049272 |
| PCDH9 | 739.9991 | 1.307624 | 0.297472 | 4.395796 | 1.10E-05 | 0.001341 |
| CEBPE | 12.53574 | 1.181929 | 0.35641 | 3.316203 | 0.000912 | 0.029648 |
| TCL1A | 693.7578 | 1.207355 | 0.337209 | 3.580431 | 0.000343 | 0.015858 |
| SLC12A1 | 18.82453 | 2.992647 | 0.866604 | 3.453303 | 0.000554 | 0.021804 |
| ITGA11 | 14.60666 | 1.71077 | 0.526992 | 3.246293 | 0.001169 | 0.035792 |
| MMP25 | 49.14102 | 1.639216 | 0.460018 | 3.563376 | 0.000366 | 0.016526 |
| ADGRG3 | 43.51695 | 1.658063 | 0.500767 | 3.311043 | 0.000929 | 0.029969 |
| SLC47A1 | 18.02844 | 1.315102 | 0.388602 | 3.384189 | 0.000714 | 0.025345 |
| CD79B | 466.9094 | 1.114678 | 0.230922 | 4.827085 | 1.39E-06 | 0.000297 |
| GRIN2C | 26.24232 | -2.18728 | 0.703057 | -3.11109 | 0.001864 | 0.048347 |
| USP36 | 463.6077 | -1.04313 | 0.333444 | -3.12835 | 0.001758 | 0.046384 |
| PTPRS | 69.94384 | -1.61753 | 0.31845 | -5.07936 | 3.79E-07 | 0.000129 |
| CYP4F3 | 64.70044 | 2.488839 | 0.751098 | 3.313599 | 0.000921 | 0.029849 |
| FFAR2 | 68.6067 | 1.29943 | 0.332798 | 3.904561 | 9.44E-05 | 0.006118 |
| CEACAM3 | 18.40452 | 2.126563 | 0.489479 | 4.344542 | 1.40E-05 | 0.001649 |
| ZNF404 | 34.87424 | 1.382861 | 0.218838 | 6.319114 | 2.63E-10 | 7.89E-07 |
| ZNF112 | 8.951579 | 1.43209 | 0.461361 | 3.104058 | 0.001909 | 0.048641 |
| ZNF331 | 518.5687 | -1.20543 | 0.217858 | -5.53312 | 3.15E-08 | 2.65E-05 |
| ID1 | 71.29617 | -1.31204 | 0.280589 | -4.67604 | 2.92E-06 | 0.00048 |
| SNAI1 | 221.4974 | -1.04649 | 0.333285 | -3.13993 | 0.00169 | 0.04531 |
| KCNG1 | 19.55411 | 1.351499 | 0.331954 | 4.071346 | 4.67E-05 | 0.003692 |
| ADAMTS5 | 16.25173 | 1.15683 | 0.362695 | 3.189541 | 0.001425 | 0.04084 |
| KCNJ15 | 70.35634 | 2.759925 | 0.529397 | 5.213337 | 1.85E-07 | 7.33E-05 |
| SOWAHD | 19.72473 | 1.061396 | 0.290782 | 3.650139 | 0.000262 | 0.013288 |
